# Novel Gene Signatures Predicting Primary Non-response to Infliximab in Ulcerative Colitis: Development and Validation Combining Random Forest With Artificial Neural Network

**DOI:** 10.3389/fmed.2021.678424

**Published:** 2021-09-28

**Authors:** Jing Feng, Yueying Chen, Qi Feng, Zhihua Ran, Jun Shen

**Affiliations:** ^1^Key Laboratory of Gastroenterology and Hepatology, Ministry of Health, Division of Gastroenterology and Hepatology, Inflammatory Bowel Disease Research Center, Shanghai, China; ^2^Ren Ji Hospital, Shanghai Jiao Tong University School of Medicine, Shanghai, China; ^3^Shanghai Institute of Digestive Disease, Shanghai, China; ^4^Department of Radiology, Ren Ji Hospital, Shanghai Jiao Tong University School of Medicine, Shanghai, China

**Keywords:** ulcerative colitis, infliximab, predictive model, machine learning, primary non- response

## Abstract

**Background:** While infliximab has revolutionized the treatment of ulcerative colitis, primary non-response is difficult to predict, which limits effective disease management. The study aimed to establish a novel genetic model to predict primary non-response to infliximab in patients with ulcerative colitis.

**Methods:** Publicly available mucosal expression profiles of infliximab-treated ulcerative colitis patients (GSE16879, GSE12251) were utilized to identify potential predictive gene panels. The random forest algorithm and artificial neural network were applied to further screen for predictive signatures and establish a model to predict primary non-response to infliximab.

**Results:** A total of 28 downregulated and 2 upregulated differentially expressed genes were identified as predictors. The novel model was successfully established on the basis of the molecular prognostic score system, with a significantly predictive value (AUC = 0.93), and was validated with an independent dataset GSE23597 (AUC = 0.81).

**Conclusion:** Machine learning was used to construct a predictive model based on the molecular prognostic score system. The novel model can predict primary non-response to infliximab in patients with ulcerative colitis, which aids in clinical-decision making.

## Introduction

While the exact pathogenesis of ulcerative colitis (UC) remains unclear, factors including genetic predisposition, environmental factors, intestinal barrier defects, and dysregulation of the immune system all contribute to the disease (1, 2). Five-amino salicylates, corticosteroids, and azathioprine are conventionally used to induce and maintain clinical remission based on the severity and location of UC (3). Nevertheless, the clinical benefits of these traditional therapeutic drugs are limited due to their lack of specificity.

Infliximab (IFX), a monoclonal antibody against human tumor necrosis factor alpha (TNF-α), has revolutionized the treatment of UC. At present, IFX is generally used for moderate to severe UC, with the advantage of promoting mucosal healing, reducing the probability of surgery, and improving the prognosis (4). However, the response rate to IFX differs among patients. It has been reported that up to 30% of patients show primary non-response (PNR) to IFX, which means they receive no clinical benefit from IFX and effective disease treatment is often delayed (5, 6). Therefore, it is important to construct a reliable model to predict non-response to IFX in the early stages of the disease.

Currently, there are few effective tools able to accurately predict PNR due to the complexity of the IFX treatment mechanism. Rapid advances in the field of bioinformatics offer new approaches for predictions with clinical application in addition to therapeutic drug monitoring, serological antibodies, and C-reactive protein levels. Detecting genetic signatures in array data is a robust way to predict clinical response based on the hypothesis that single nucleotide polymorphisms (SNPs) related to the pathogenesis of disease or mechanism of drug action may determine the relationship between genes and drug therapeutic effect (7).

Machine learning techniques, including random forest (RF) and artificial neural network (ANN), have been successful in biomarker discovery and in studies spanning multiple disease types (8–10). With the development of machine learning, the most significant differentially expressed genes (DEGs) can be selected and converted to statistical models to guide clinicians to reasonable and effective therapeutic options (11). In our previous study, we identified the TNFRSF1B SNP variation as a predictor for secondary non-response to IFX in Crohn's disease. However, a comprehensive analysis of the genetic predictors of IFX response in patients with UC is still lacking. Thus, the aim of this study was to develop and validate a genetic model based on machine learning to predict IFX PNR in patients with UC.

## Materials and Methods

### Study Design and Processing

**Figure 1:**
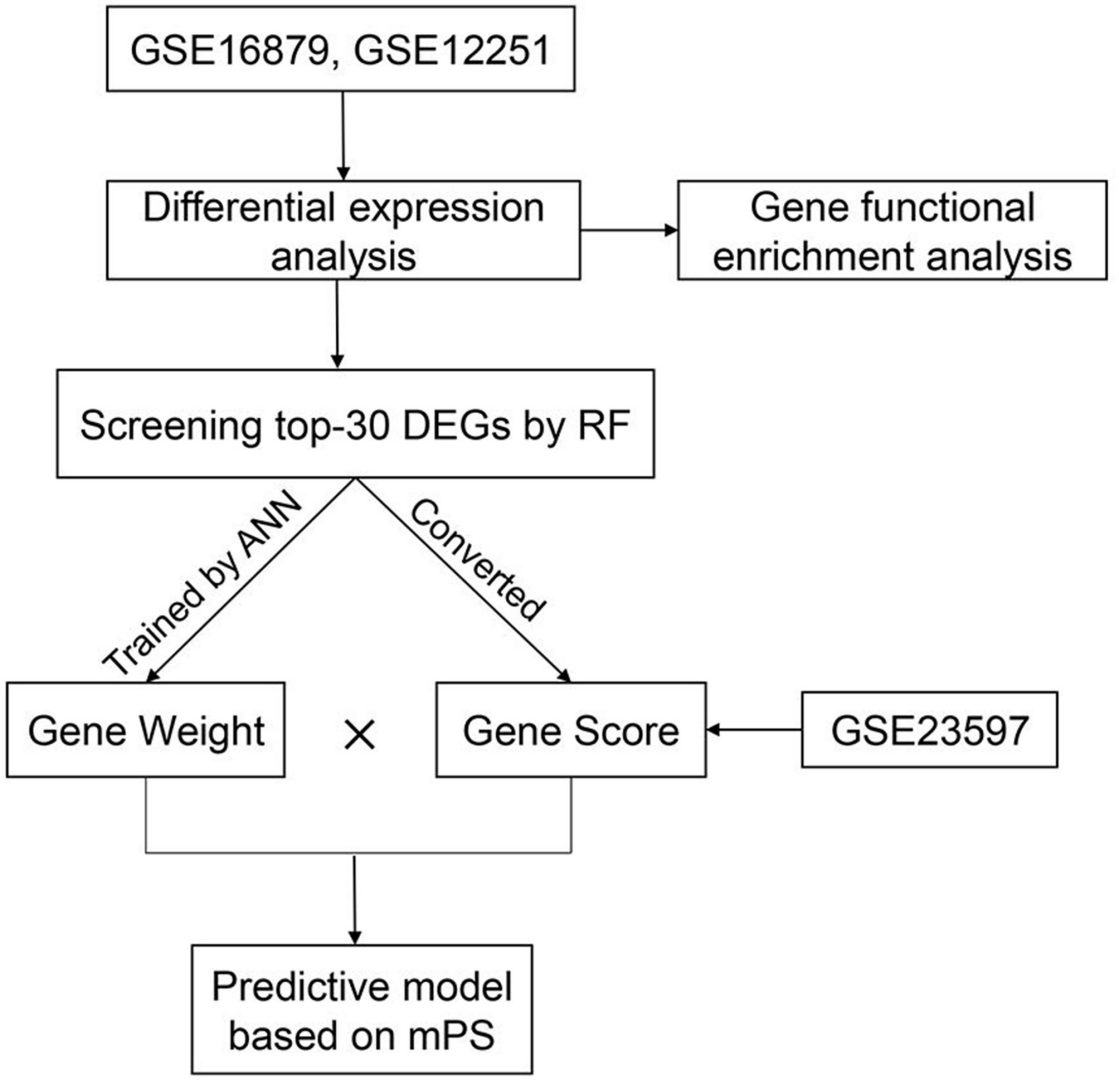
The study flowchart.

In order to predict IFX PNR prior to treatment, three sets of mucosal array profiles at the baseline (week 0) in IFX-treated UC patients were employed in this study (GSE16879, GSE12251, and GSE23597). The training datasets comprised GSE16879 and GSE12251, and GSE23597 was selected as the validation dataset. In this study, whether patients responded to IFX was defined according to the Mayo endoscopic subscore and histological score for UC. The therapeutic effect of IFX in all study participants was evaluated within 14 weeks of starting treatment; PNR was diagnosed if patients showed no endoscopic improvement in this time. RF was used to further screen the top-30 DEGs that contributed the most to the prediction of PNR to IFX in UC patients. Subsequently, gene expression scores were calculated according to the expression data of DEGs from all samples. The weight values of the top-30 DEGs were attained by developing an ANN model. Then, we used the weight values and gene expression scores to build a molecular prognostic score (mPS) system, and GSE23597 was used as a validation dataset to prove the efficacy of the novel predictive model. The study flowchart is shown in Figure 1. Institutional review board approval is not needed for this study.

### Datasets and Identification of DEGs

The raw data of the datasets used in our study were downloaded from the Gene Expression Omnibus (GEO; https://www.ncbi.nlm.nih.gov/geo/). These three datasets were derived from the same microarray platform, GPL570 [(HGU133_Plus_2) Affymetrix Human Genome U133 Plus 2.0 Arrays]. Analysis and verification were conducted on the expression profiles of IFX-treated UC patients at the baseline, which were extracted from diseased rectal biopsies within a week prior to the first intravenous infusion of 5 mg infliximab per kg body weight (12, 13). According to the clinical information provided by the datasets, these patients were classified as responders and non-responders.

The raw data were processed and standardized using R software (version 4.0.1). The datasets for gene expression were normalized using the RMA algorithm. Multiple probes associated with the same gene were deleted and summarized for further analysis. The Limma package was used to remove the batch effect by building linear models, and to identify DEGs. The significant DEGs of the training dataset were identified with the threshold: false discovery rate <0.05, and |log2 (Fold Change) |>1. A volcano plot was generated to visualize the DEGs.

### Gene Ontology and Pathway Enrichment Analysis

Metascape (http://metascape.org) was used to perform pathway enrichment and biological process annotation, providing comprehensive and detailed information for each gene (14). In this study, Metascape was used to carry out gene ontology and pathway enrichment analysis in order to identify the functional biological terms and signaling pathways of significant DEGs in the training dataset. Only terms with *P* <0.01 and a count of enriched genes ≥3 were considered significant. All the significant terms were then grouped into clusters based on their membership similarities, and the most enriched term was chosen to represent the cluster.

### Screening DEGs Predicting PNR to IFX With Machine Learning

The Random Forest package in R version 4.0.1 was applied to further screen out 30 DEGs that contributed the most to the prediction of PNR to IFX in UC patients. The top 30 is a common selection criterion based on the algorithm requirements of RF packages and has been widely used in similar studies (15, 16). Subsequently, the expression data of the 30 DEGs were converted into a score table named “Gene Score,” according to the diagnosis of UC (17). The specific conversion rules are as follows: If the expression value of an upregulated gene in a certain sample is higher than the median expression value of the gene in all samples, its expression value will be converted to 1, otherwise 0. If the expression value of a downregulated gene is higher, its expression value will be converted to 0, otherwise 1. As for the therapeutic effect of IFX in UC patients, responders are converted to 1 and non-responders are converted to 0. Above all, the Gene Score is composed of 46 lines of samples, 30 columns of DEGs, and column of response to IFX (response/non-response).

Finally, we used the Python-based Keras library to establish an ANN forecast model. The therapeutic result of IFX was designated as y, and the Gene Score of each of the top-30 DEGs was designated as x. The ANN was composed of one input layer, one hidden layer, and one output layer. In the hidden layer, we set ten hidden nodes and exploited rectified linear unit as an activation function. In the output layer, we set two nodes (response/non-response) and the activation function of each node was a softmax function. The cross-entropy error was set as a loss function and the Adam method was used to optimize the value of each weight. After training, we selected the maximum weight value of a certain DEG in the hidden layer named “Gene Weight” (18).

### Development and Validation of the Predictive Model

The construction of the model to predict PNR to IFX in UC patients was based on the mPS system. As an innovative scoring system, mPS was created in 2019, and is effective in the prediction of overall survival of breast cancer patients and the diagnosis of UC (17, 18). The mPS of each sample was calculated by summation of “Gene Score” × “Gene Weight” for all top-30 DEGs (18).

The array data in GSE23597 were used to validate the effectiveness of the mPS scoring system based on the training dataset. According to the conversion rules, we obtained an updated “Gene Score,” and calculated the summation of “Gene Score” × “Gene Weight.” The area under the receiver operating characteristic curve (AUC) was used to evaluate the predictive value of this model, and it was calculated using the ROCR package in R (version 4.0.1). If the AUC value was higher than 0.8, it was considered an excellent discrimination. If the AUC value was higher than 0.9, it was considered an outstanding discrimination (19).

## Results

### Determination of Sample Group

UC patients initially treated with IFX induction therapy in GSE16879 and GSE12251 were enrolled into the training dataset. The expression profiles at the baseline (week 0) were used for further analysis. The response to IFX was defined as complete mucosal healing with a Mayo endoscopic subscore of 0 or 1 and a grade 0 or 1 on the histological score for UC (12, 20). Non-responders were patients who did not achieve healing, although some presented with minor endoscopic or histologic improvement (12, 20). The response to IFX was assessed 4 weeks after the first IFX therapy in the GSE16879 dataset and 8 weeks in the GSE12251 dataset.

The validation dataset was UC patients initially treated with IFX in GSE23597, and the response to IFX was assessed 8 weeks after the first IFX therapy (13, 21). The specific information of the training and validation datasets we selected is shown in Table 1.

**Table 1:** The information of training/validation datasets.

**Dateset ID**	**Platform**	**UC**	**response**	**Non-response**
GSE16879	GPL570	24	8	16
GSE12251	GPL570	22	12	10
GSSE23597	GPL570	28	21	7

### Identification of DEGs

**Figure 2:**
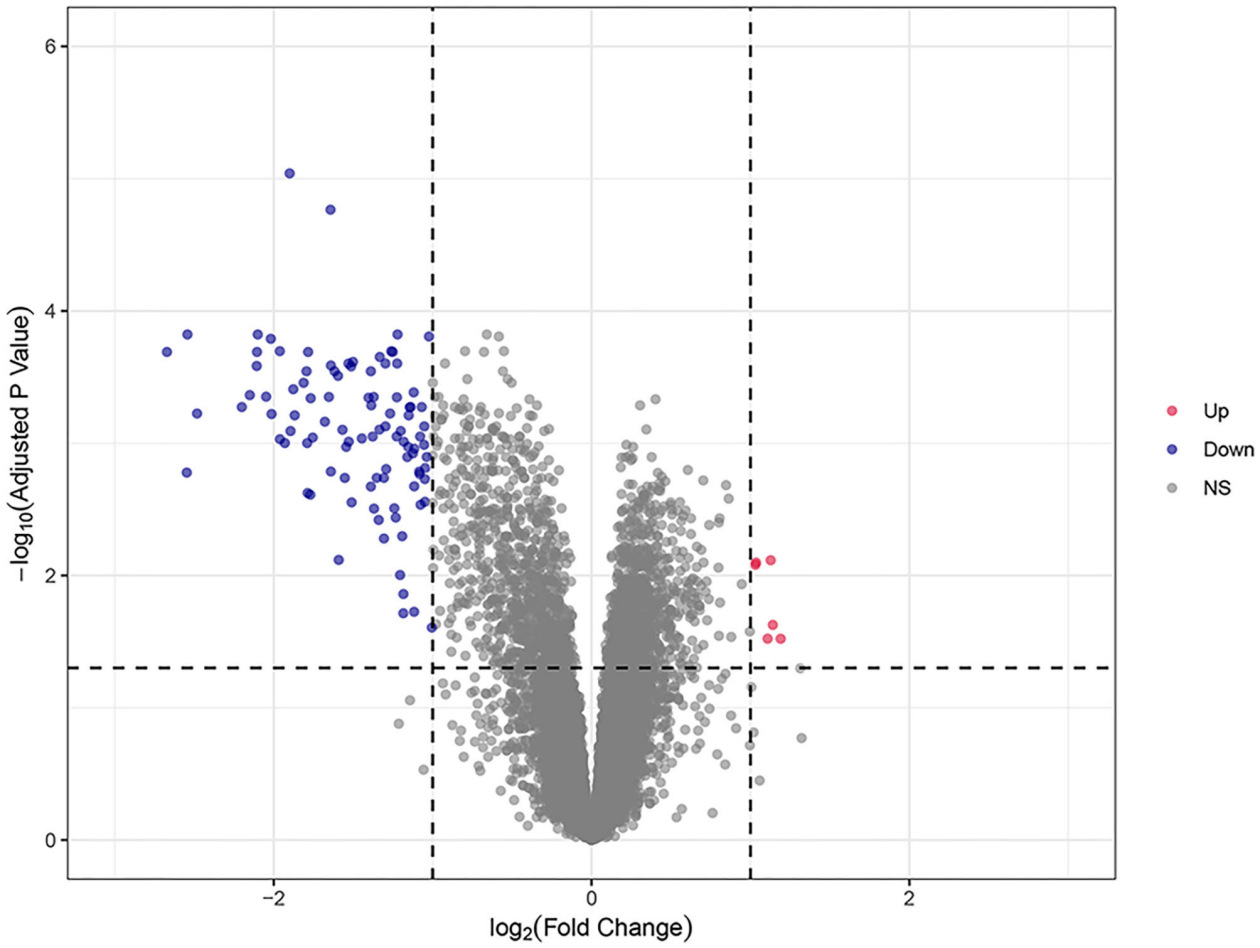
The volcano plots of all DEGs in the training dataset. Blue and red spots represent downregulated and upregulated genes, respectively. A clear demarcation can be identified between upregulated genes and downregulated genes.

A total of 104 DEGs were identified in the training dataset (Figure 2). The expression status of all DEGs is shown in the volcano plot, from which we can observe that most of the DEGs were downregulated in responders and upregulated in non-responders. Only six of them (HEPACAM2, C10orf99, HSD11B2, ADH1C, PKIB, and CHP2) were upregulated in responders and downregulated in non-responders.

### Functional Enrichment Analysis of DEGs in the Training Dataset

**Figure 3:**
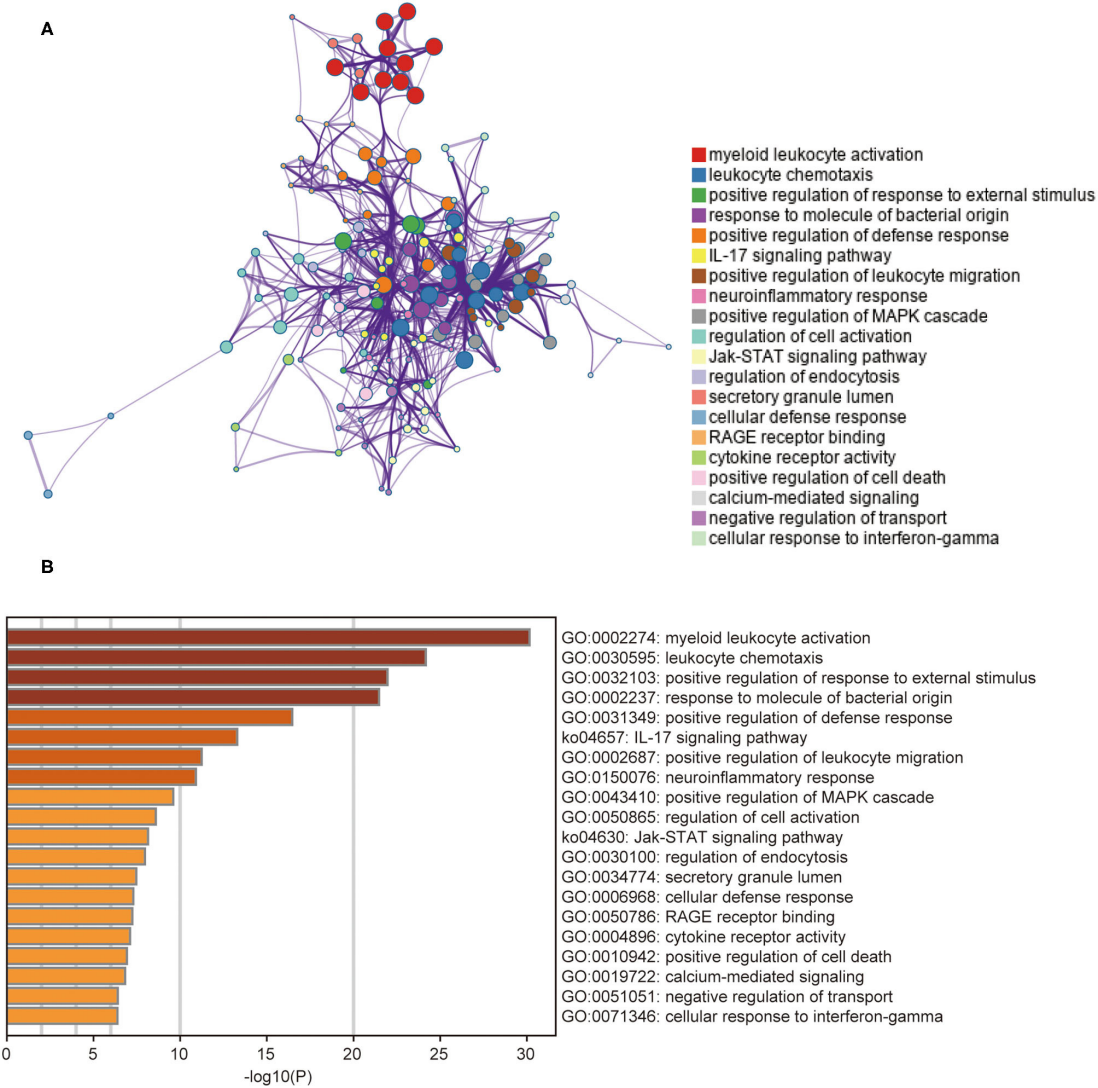
The results of gene functional enrichment analysis. **(A)** The network of the top 20 clusters of enriched terms. Cluster identity is represented by color, the similarity score is represented by the thickness of the edge, and terms with a similarity score > 0.3 are linked by an edge. **(B)** Heat map of the top 20 clusters. Cluster identity is represented by color; the smaller the *P*-value the deeper the color.

To further understand the functions and metabolic pathways associated with these DEGs, enrichment analysis was performed using Metascape. The Metascape analysis showed the top 20 clusters in which DEGs were significantly enriched (Figure 3). The top enriched gene ontology terms in biological process were “myeloid leukocyte activation,” and “leukocyte chemotaxis.” Interestingly, in KEGG pathway analysis, DEGs were mainly involved in the “IL-17 signaling pathway” and “JAK-STAT signaling pathway.” These enriched terms and pathways were upregulated in non-responders, and their inhibitors can be considered as alternative treatment strategies for these patients.

### Top 30 DEGs Screened by RF

**Figure 4:**
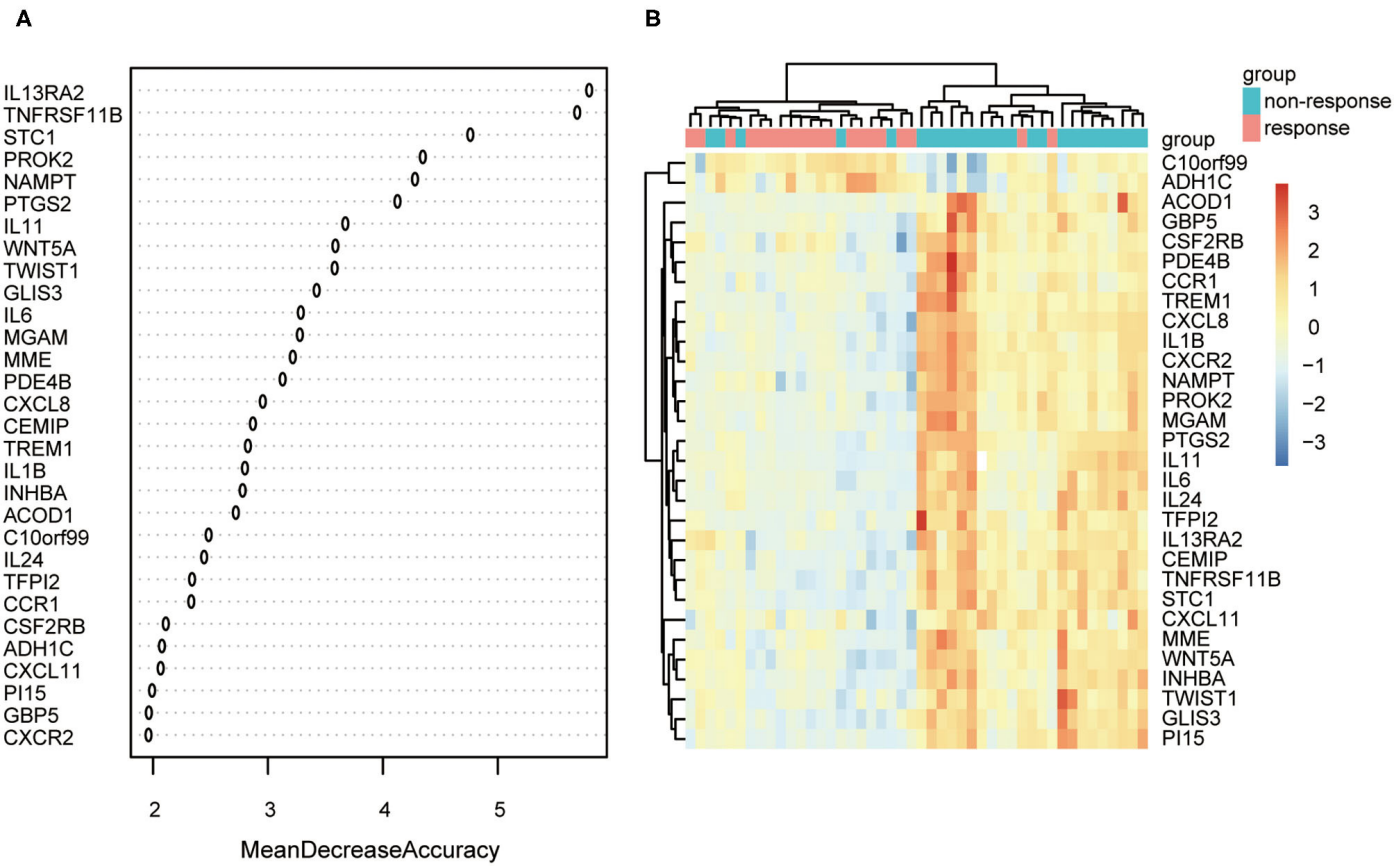
The results of the top 30 genes screened by random forest. **(A)** The importance of the top 30 genes ranked by mean decrease of accuracy. **(B)** Heat map of the top 30 genes.

The expression data of 104 DEGs were included in the RF classifier (Figure 4A). The top 3 response-related genes were IL13RA2, TNFRSF11B, and STC1. Except for C10orf99 and ADH1C, the other 28 genes were downregulated in responders and upregulated in non-responders. The heat map (Figure 4B) shows the expression status of the top 30 DEGs.

### ANN-based Establishment of the mPS

**Figure 5:**
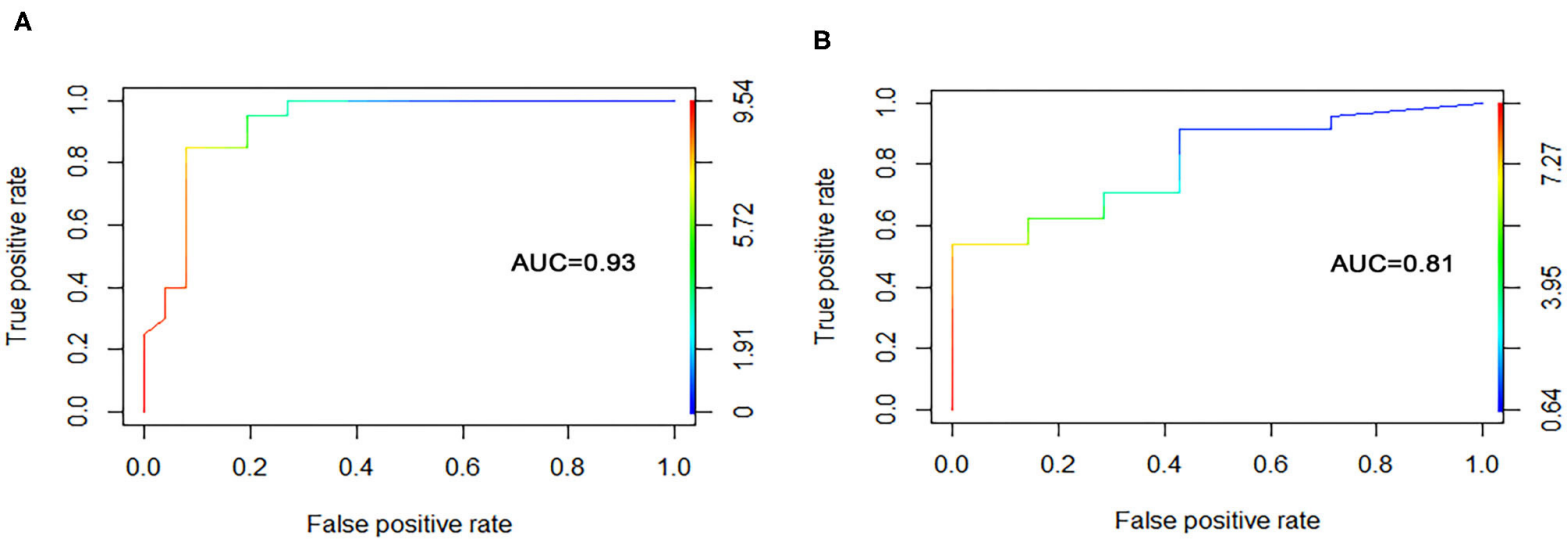
The ROC curve of the predictive model. **(A)** Training dataset. **(B)** Validation dataset.

The ANN algorithm was used to optimize the weight value of each gene after the expression data of the 30 DEGs were converted into “Gene Score.” The Gene Weight of each gene is shown in Table 2. The mPS was calculated by summation of “Gene Score” × “Gene Weight” for all 30 DEGs. Then, we set the mPS of 46 samples as predicted values and set the response of UC patients to IFX as true values. Using the ROCR package (R version 4.0.1), the AUC of our model was found to be 0.93, indicating that our model achieved outstanding predictive power (Figure 5A).

**Table 2:** The “Gene Weight” of top 30 DEGs in the training dataset.

**Gene symbol**	**weight**	**Gene symbol**	**weight**
IL13RA2	0.3404	CEMIP	0.3602
TNFRSF11B	0.2082	TREM1	0.3959
STC1	0.3198	IL1B	0.3385
PROK2	0.3326	INHBA	0.2298
NAMPT	0.3178	ACOD1	0.4049
PTGS2	0.0912	C10orf99	0.3389
IL11	0.342	IL24	0.2098
WNT5A	0.3351	TFPI2	0.4268
TWIST1	0.3171	CCR1	0.3559
GLIS3	0.3627	CSF2RB	0.2966
IL6	0.4434	ADH1C	0.0852
MGAM	0.3457	CXCL11	0.4086
MME	0.4478	PI15	0.2851
PDE4B	0.2839	GBP5	0.1814
CXCL8	0.3087	CXCR2	0.1764

### Validation of the Predictive Model

An independent dataset (GSE23597) was used to test whether the model we built could predict the therapeutic effect of IFX in the training dataset as well as any other independent cohort. Similarly, we used RF to screen out the top 30 DEGs of the validation set. These DEGs were the same as those of the training set, demonstrating the scalability and robustness of RF. Then, we calculated the “Gene Score” and mPS of GSE23597 in the same way as the training set. The AUC of the validation model was 0.81, confirming the validity and stability of our model (Figure 5B).

## Discussion

Infliximab has demonstrated efficacy in the treatment of moderate to severe UC, achieving mucosal healing even in steroid-refractory patients (21). However, PNR to IFX emerges due to genetic factors, as it relates to disease pathogenesis and the mechanism of action of this type of therapy (22). It is necessary to develop a simple and effective method to rapidly identify UC patients who exhibit IFX PNR. In the present study, an innovative model was established and validated to predict PNR to IFX in UC patients on the basis of machine learning and a new clinical prediction scoring system called “mPS,” which has already proven useful in the prediction of malignant diseases.

Previous research on patients with inflammatory bowel disease (IBD) has identified several genes (IL13RA2, TNFRSF11B, STC1, IL-6, and IL-11) that constitute potential biomarkers that can identify patients with limited response to IFX, which is consistent with our study (12, 23). However, a systematic, robust, and reliable approach that can be applied to clinical decision-making has not yet been developed. In this study, we combined the strengths of machine learning techniques and mPS, not only to improve the statistical power of our predictive model, but also to transfer theoretical predictive gene panels for use in routine clinical practice. The main advantages of RF include its relatively good accuracy, robustness, and ease of use, allowing it to recognize the discriminative genes with the highest possible accuracy (24). High fault and failure tolerance, scalability, and consistent generalization ability are merits of ANN, making the model more stable and reliable (25). Therefore, our model is equipped with outstanding predictive power (AUC = 0.93) compared to another genetic model built by Bruke et al. (AUC = 0.87) (7). In addition, the mPS scoring system has proven to be simple, cost-effective, and excellent in recognizing heterogeneity among different subtypes (18). It converted complex gene expression values into simple clinical scores, so that the model can facilitate doctors in formulating a reasonable, personalized, and economical IFX regimen for UC patients.

Gene enrichment analysis of DEGs showed that most of the screened DEGs were involved in myeloid leukocyte activation and leukocyte chemotaxis. It has been reported that an increased abundance of leukocytes in non-responders promotes an increase in inflammatory macrophages, which secrete proinflammatory cytokines, including TNF-α (26). Therefore, vedolizumab, an inhibitor of α4β7 that blocks leukocyte traffic to the gut, can be used to treat non-responders as an alternative therapy. We also identified several pathways, such as the “IL-17 signaling pathway” and “JAK-STAT signaling pathway,” which were significantly enriched in the non-response group. IL-17 is involved in the induction and persistence of IBD mucosal inflammation (27). The JAK-STAT pathway is the main signal mechanism for a variety of cytokines and growth factors. It transmits extracellular cytokine stimulation signals to the nucleus, coordinates appropriate cellular responses through target gene expression, and is closely related to human inflammatory diseases (28–30). Inhibitors of Janus kinases, such as Tofacitinib or Filgotinib, can thus be considered alternative treatment options for IFX non-responders. Although increased level of IL-17 was detected in intestinal mucosa of patients with Crohn's disease and ulcerative colitis, IL-17 might be a predictor or protective factor for intestinal inflammation rather than therapeutic target due to the ineffectiveness in clinical trials of Crohn's disease (31, 32). The correct interpretation of gene enrichment analysis not only contributes to understanding the molecular mechanism of PNR, but also provides a scientific basis for the research and development of new alternative drugs.

Moreover, the gene signatures identified in this study seem to be predictive of secondary loss of response (LOR) to IFX as well. Although secondary LOR is commonly attributed to the formation of anti-TNF antibody, it does share several common risk factors with PNR such as high inflammatory burden, male gender and so on (33). In addition, our previous work has confirmed the value of TNFRSF1B in the prediction of secondary LOR to IFX in Crohn's disease, indicating that other predictive genes of PNR may be equally applicable to secondary LOR (34). In contrast to PNR, secondary LOR is generally accompanied by a lower serum level of IFX due to high levels of anti-TNF antibody stimulated by frequent infusion of IFX (35). Proactive monitoring drug concentration in patients with predictive gene signatures may be a good method to optimize therapy and prevent the occurrence of secondary LOR.

However, our study has several limitations. First, although our predictive model performed satisfactorily on the training and validation datasets, the sample size used to develop and validate the predictive model was relatively small. Second, the model was validated on a dataset from GEO. To make the model plausible, further bench experimental verification should be carried out. Third, the genetic model we built only applies to the identification of primary non-responders from responders in UC. Whether this model can be applied to predict the PNR of CD patient needs to be further vitrificated.

## Conclusions

We established a predictive model based on machine learning techniques and an mPS scoring system that could be used to predict which UC patients will exhibit PNR to IFX, and validated this model with an independent cohort from the GEO database. Our study provides clinicians with a new treatment strategy that can improve therapeutic decision-making. The predictive genes and corresponding pathways identified in this model should be further studied to explore the molecular mechanisms underlying patient response to IFX.

## Data Availability Statement

The datasets presented in this study can be found in online repositories. The names of the repository/repositories and accession number(s) can be found in the article/supplementary material.

## Author Contributions

JF analyzed and interpreted the high throughput data, prepared, and wrote the manuscript. ZR and JS designed and drafted the work. YC helped analyse part of the data. YC and QF edited and revised manuscript. All authors read and approved the final manuscript.

## Funding

This work was supported by the National Natural Science Foundation of China (grant nos. 81770545, 81701746, and 81670497) and the MDT Project of Clinical Research Innovation Foundation, Renji Hospital, School of Medicine, Shanghai Jiao Tong University (grant no. PYI-17-003).

## Conflict of Interest

The authors declare that the research was conducted in the absence of any commercial or financial relationships that could be construed as a potential conflict of interest.

## Publisher's Note

All claims expressed in this article are solely those of the authors and do not necessarily represent those of their affiliated organizations, or those of the publisher, the editors and the reviewers. Any product that may be evaluated in this article, or claim that may be made by its manufacturer, is not guaranteed or endorsed by the publisher.

## Abbreviations

UC: Ulcerative Colitis
IFX: Infliximab
TNF-α: Tumor necrosis factor alpha
PNR: Primary Non-Response
SNPs: Single Nucleotide Polymorphisms
RF: Random Forest
ANN: Artificial Neural Network
DEGs: Differentially expressed genes
mPS: molecular Prognostic Score
AUC: The area under the receiver operating characteristic curve
LOR: Loss of response.
